# Erroneous formulation of delayed-release omeprazole capsules: alert for importing countries

**DOI:** 10.1186/s40360-017-0138-5

**Published:** 2017-05-03

**Authors:** Mohammad Sofiqur Rahman, Naoko Yoshida, Hirohito Tsuboi, Tep Keila, Tey Sovannarith, Heng Bun Kiet, Eav Dararth, Theingi Zin, Tsuyoshi Tanimoto, Kazuko Kimura

**Affiliations:** 1 Drug Management and Policy, Kanazawa University, Kanazawa, Japan; 2 National Health Product Quality Control Center, Ministry of Health, Phnom Penh, Cambodia; 3 Department of Drugs and Food, Ministry of Health, Phnom Penh, Cambodia; 4 Department of Food and Drug Administration, Ministry of Health and Sports, Naypyidaw, Myanmar; 5 Faculty of Pharmaceutical Sciences, Doshisha Women’s University, Kyoto, Japan

**Keywords:** Substandard, Omeprazole, Enteric coating, Myanmar, Cambodia

## Abstract

**Background:**

Poor drug quality is a matter of serious concern, especially in countries where drug regulation and law enforcement are constrained by limited resources. This study was carried out to investigate the cause of quality failure of omeprazole in Cambodia in 2010 and Myanmar in 2014.

**Methods:**

We conducted pharmacopoeial quantity, content uniformity and dissolution tests of 156 samples of omeprazole capsules collected in Cambodia in 2010 and Myanmar in 2014. High failure rates were found, especially in dissolution testing, and detailed investigation of several unacceptable samples was carried out by means of in-vitro dissolution profiling, scanning electron microscopy (SEM) and X-ray computed tomography (X-ray CT) to identify the cause of failure.

**Results:**

Dissolution profiling with and without the acid stage showed that acid caused premature omeprazole release, indicating that the enteric coating of the omeprazole granules was ineffective. SEM examination of two failed samples revealed cracked and broken granules mixed with apparently intact omeprazole granules in the capsule. X-ray CT examination indicated that some granules of failed samples completely lacked enteric coating, and others had incomplete and non-uniform enteric coating or malformation.

**Conclusions:**

Omeprazole capsules collected in Myanmar and Cambodia showed high failure rates in pharmacopoeial tests, especially dissolution tests. Some samples were found to have ineffective or absent enteric coating of the granules, resulting in premature dissolution and degradation in acidic conditions. This is a potentially serious public health issue that needs to be addressed by regulatory authorities in Cambodia and Myanmar, possibly through a collaborative initiative with manufacturers.

## Background

There is considerable evidence that the incidence of falsified and substandard medicines is increasing, particularly in middle- and lower-income countries [1–3]. There have been many well-established instances of spurious, falsely labeled, falsified or counterfeit (SFFC) medicines in recent years [4–10]. In addition, substandard medicines, which are prepared by legitimate manufacturers but fail to meet pharmacopoeial requirements, also constitute an enormous public health problem [11–15]. On-going surveillance seems essential.

The substituted benzimidazoles are a class of anti-secretory compounds that suppress gastric acid secretion by inhibition of the H^+^/K^+^-ATPase enzyme system at the secretory surface of gastric parietal cells [16, 17]. Among them, omeprazole, 5-methoxy-2-[[(4-methoxy-3,5-dimethyl-2-pyrinylmethyl-sulfinyl)-1H-benzimidazole, is a basic compound that acts as a proton pump inhibitor, and is used in the treatment of acid reflux and heartburn [18]. It is acid-labile, being degraded rapidly in aqueous solution at low pH [19, 20]. Pre-formulation studies confirmed that it is susceptible to moisture, heat and acidic solvents [21, 22]. Therefore, to avoid degradation of omeprazole by acid in the stomach, the drug must be enteric-coated [23, 24]. Consequently, omeprazole dosage forms are prepared and marketed in an enteric-coated form that allows the omeprazole core to be specifically released and dissolved in the duodenum (pH > 5) or terminal ileum where the pH is about 6.8 to 7.5 [25].

Despite increasing attention to the quality of medicines for communicable diseases, focus on medicines for non-communicable diseases remains inadequate. In 2010, quality test results of omeprazole in Cambodia indicated that more inspection and monitoring of medicines for non-communicable diseases is necessary [6]. The availability of falsified and substandard medicines in Myanmar was reported by WHO in 1999 [15], but since then there has been no systematic survey in the country, and the current situation is unclear, except for sporadic reports of falsified medicines. Based on our experience in Cambodia during 2006-2013 [6], where we encountered various poor quality (mostly substandard) medicines, omeprazole was chosen as one of the target medicines for investigation in Myanmar, in consultation with the Department of Food and Drug Administration (FDA), Myanmar. Among 156 samples of omeprazole capsules collected in Cambodia in 2010 and Myanmar in 2014, we found high failure rates in pharmacopoeial tests, especially dissolution tests. This is broadly consistent with other reports of substandard drugs in Cambodia [26, 27].

The aim of the present study was to establish the cause of the high failure rate of omeprazole capsules from the two countries in dissolution tests by means of detailed evaluation of the in-vitro dissolution profile, as well as scanning electron microscopy (SEM) and X-ray computed tomography (X-ray CT) examinations.

## Methods

### Materials

United States Pharmacopeia (USP) reference standard omeprazole was procured from USP Convention. Authentic omeprazole standard capsules (Losec) were provided by AstraZeneca. Lansoprazole (internal standard) was from Sigma Aldrich (India). NaH_2_PO_4_.2H_2_O, Na_2_HPO_4_, Na_3_PO_4_, KH_2_PO_4_ and other chemicals of reagent grade were purchased from Nacalai Tesque Inc. (Kyoto, Japan). Distilled water was used for the preparation of HPLC eluents.

The investigational samples consisted of 154 samples of hard gelatin capsules containing 20 mg of omeprazole in enteric-coated pellets and 2 tablet samples purchased from different drug stores in Cambodia in 2010 and Myanmar in 2014. In Cambodia, samples were collected from pharmacies, depots, wholesaler and outlets of Phnom Penh, Svay Rieng and Kandal provinces. In Myanmar, collected samples were from pharmacy, hospital, and wholesalers of Yangon region. These samples of different serial and batch number were imported to Cambodia and Myanmar from 53 different manufacturers in seven countries. Samples were stored below 25 °C after collecting and all the quality analysis of the samples was finished before the expiration date of the samples. After quality-testing as required by the indicated pharmacopoeia, we selected five samples for further investigation based on the gravity of their failure in the dissolution test.

### HPLC determination of omeprazole

To assess the pharmaceutical quality of the omeprazole samples, the active ingredient was detected by a simple reverse phase high performance liquid chromatography (HPLC). HPLC was run on a Phenomenex Gemini NX C18 column (150 × 4.6 mm), with a Prominence HPLC Photo Diode Array Detector (SPD-20A/20AV Series; Shimadzu, Kyoto, Japan). The temperature of the column oven was set to 25 °C. Elution buffer was prepared by dissolving 1.17 g NaH_2_PO4.2H_2_O and 1.06 g Na_2_HPO_4_ in 1000 ml of water; the pH was adjusted to 6.8. The column was eluted with a mixture of phosphate buffer and acetonitrile (60:40) at a flow rate of 0.5 ml/min. Detection wavelength and injection volume were 302 nm and 10 μL, respectively for British Pharmacopoeia (BP) samples. For USP samples, the flow rate was 1 ml/min, detection wavelength was 280 nm, and injection volume was 10 μL. The system suitability for the analysis of omeprazole was verified according to USP 37. The limit of detection (LOD) and limit of quantification (LOQ) of omeprazole was determined by the slope of the calibration curve and the standard deviation of responses. For each linearity solution of 5 different concentrations, average area of the multiple injections (n = 3) were taken and graph of concentration in μg/ml (X-axis) versus peak area response (Y-axis) was plotted. LOD and LOQ concentrations of omeprazole were determined on the basis of equation,

$$ \mathrm{Limit}\ \mathrm{of}\ \mathrm{Detection} = 3.3\times \sigma /\mathrm{S}, $$

and

$$ \mathrm{Limit}\ \mathrm{of}\ \mathrm{Quantification} = 10\times \sigma /\mathrm{S} $$

Where, σ = the standard deviation of the response

S = the slope of the calibration curve

A linear relationship between the peak area and concentration of each reference standard was observed within the range of 25–200% of the active ingredient (r2 = 0.999–1.000), and the assay was performed within that range. In addition, the method was validated as being repeatable and accurate (*n* = 6).

### Omeprazole quantity and content uniformity tests

Quantity and content uniformity tests of omeprazole samples were carried out according to the modified method of BP 2010 and 2015 [28, 29] or USP 34 and 37 [30, 31] as indicated in the package insert or on the outer package of each sample. For the identification test, a chromatogram of the sample was compared with that of reference standard omeprazole. The retention time of the principal peak in the sample chromatograms was similar to that of the peak of standard omeprazole. Standard solutions were prepared by dissolving accurately weighed quantities of omeprazole (reference standard) and lansoprazole (internal standard) in the diluent to obtain solutions with concentrations of 0.2 mg/mL and 0.1 mg/mL, respectively. From these stock solutions, five diluted omeprazole solutions (0.2, 0.15, 0.1, 0.05 and 0.025 mg/mL) were prepared. The relationship between the peak area and concentration of each reference standard was linear within the range of 25–200% of the active ingredient (r^2^ = 0.999–1.000), and the quality test was performed within that range.

In the quantity test, acceptance criteria of omeprazole for BP samples were as follows: tablets/capsules contain the equivalent of not less than 95.0% and not more than 105.0% of the labeled amount of omeprazole. For USP samples, the acceptance range was between 90–110%. The acceptance value for the content uniformity test was ≤ 15. In the dissolution test, the maximum percentage of omeprazole allowed to be dissolved in the acid stage for BP samples was the average of 24 units is not more than 10% and no individual unit is not more 25% of omeprazole dissolved. For USP samples, the tolerance in the acid stage was the average of 24 units is not more than 20% of omeprazole dissolved, not more than two units are greater than 35% of omeprazole dissolved and no individual unit is greater than 45% of omeprazole dissolved. In the buffer stage, the acceptance criterion for BP samples was the average value of 24 units is equal to or greater than Q (Q = 65%), not more than 2 units are less than Q-15% & no unit is less than Q-25% (Q = 65) and for USP samples, criterion was the average value of 24 units is equal to or greater than Q (Q = 75%), not more than 2 units are less than Q-15% & no unit is less than Q-25% (Q = 75).

### Omeprazole dissolution test and examination of dissolution profile

The dissolution test was performed according to the BP or USP as indicated by the sample products. BP Samples were exposed to 700 mL phosphate buffer, pH 4.5, for 45 min in acid stage and 900 mL phosphate buffer, pH 6.8 for 45 min in buffer stage [28, 29]. USP samples were exposed to 500 mL of 0.1 N HCl for 2 h in acid stage and 900 mL of phosphate buffer, pH 6.8 for 30 min in buffer stage [30, 31]. The dissolution test was conducted with a NTR-VS 6P dissolution apparatus (Toyama Sangyo Co. Ltd., Osaka, Japan). Drug release studies were carried out by paddle method. The paddle was set to rotate at 100 rpm and the temperature was maintained at 37 ± 0.5 °C.

To examine the dissolution profile, three capsules were used. Since all the failed samples were BP samples, in the investigation with the acid stage, test samples were exposed to 700 mL phosphate buffer, pH 4.5, for 45 min. After 45 min, a 5 mL aliquot was withdrawn from each vessel for quantification by HPLC. Then 200 mL of phosphate buffer, pH 7.6, was added to adjust the final pH to 6.8 (buffer stage). In this stage, 5 mL aliquots were withdrawn from each dissolution vessel at 5, 15, 30, 45, and 60 min for quantification by HPLC. In the investigation without the acid stage, samples were exposed to the buffer stage directly without the previous acid stage, and samples were collected in the same manner as described above.

### Scanning electron microscopy

Surface morphology of omeprazole granules was characterized by means of scanning electron microscopy on a Hitachi S-3400 instrument equipped with a Hitachi E-1010 ion sputter device. A few omeprazole granules were removed from the capsule shell, mounted on a stub of metal with adhesive, coated with platinum, and observed.

### X-Ray computed tomography

X-Ray CT of the samples was conducted using an inspeXio SMX-100CT (Shimadzu) equipped with a sealed tube type micro focus X-ray generator with a maximum output of 100 kV, and a high-sensitivity image intensifier. The sample granules were positioned between the X-ray generator and the X-ray detector, and X-ray fluoroscopic data was collected from every angle by rotating the sample through 360°. Finally, computed tomographic images (CT images) were calculated from the obtained data.

### Statistical Analysis

Statistical analyses were performed using Microsoft Excel and SPSS 19.0.0 (IBM SPSS Inc. Chicago, IL, USA). Statistical differences between the experimental groups were analyzed by Student’s *t-*test. Statistical significance was evaluated at the 5% level.

## Results

### Quality analysis of collected samples

Among the 156 omeprazole samples collected, 45 (28.8%) were unacceptable in the quantity test, while 23 (14.7%) were unacceptable in the content uniformity test. In the dissolution test, 90 samples (57.7%) were acceptable and 62 (39.7%) were unacceptable (Table 1).

**Table 1:** Outline of the samples and the summary of the quality test results for omeprazole collected in Cambodia 2010 and Myanmar 2014

Country	Shop category	No. of samples (n/%)	Country of manufacturer		Quality tests	Acceptable	Unacceptable	Pending^a^
			Domestic (n/%)	Imported (n/%)		n/%	n/%	n/%
Cambodia 2010 (*n* = 91)	Pharmacy	26/28.5	2/2.2	89/97.8	Quantity	54/59.3	22/24.2	15/16.5
	Depot	45/49.5			Content Uniformity	31/34.1	14/15.4	46/50.5
	Wholesaler	8/8.8						
	Outlet	12/13.2			Dissolution	42/46.2	45/49.4	4/4.4
Myanmar 2014 (*n* = 65)	Pharmacy	35/53.8	0/0	65/100	Quantity	42/64.6	23/35.4	0/0
	Hospital	26/40			Content Uniformity	56/86.2	9/13.8	0/0
	Wholesaler	4/6.2			Dissolution	48/73.8	17/26.2	0/0

^a^Insufficient material was available for full testing

### Dissolution profile: Effect of the acid stage on drug release

**Fig. 1:**
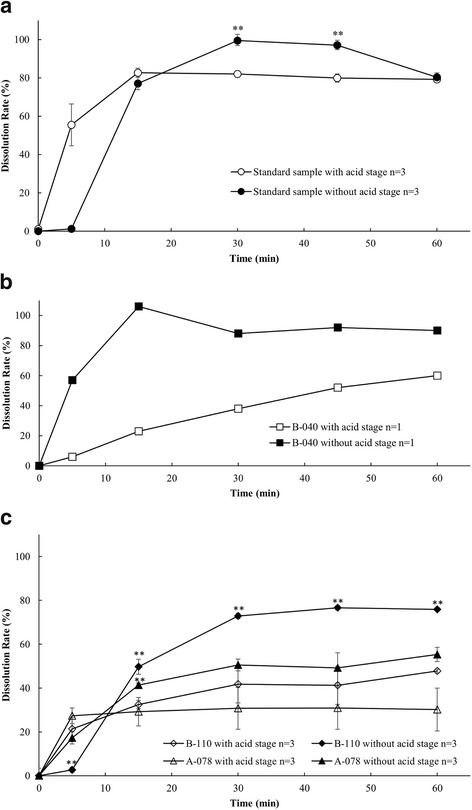
Dissolution profiles (percent release) of omeprazole in buffer stage with and without acid stage. **a** The standard sample; **b** one failed omeprazole sample (B-040) from Cambodia; **c** two failed omeprazole samples from Myanmar. Each value represents mean ± SD of three capsules except for (b) where *n* = 1. Significant differences were evaluated (**p < 0.01) comparing percent dissolution of the capsules in with and without acid stage at each time point using student’s *t*-test. For the dissolution profile of Cambodian sample only one capsule was used in each stage, the result of which was not available for statistical comparison

Figure 1 (a) shows the percent of omeprazole released in buffer from a standard sample (Losec) with and without the acid stage; in this case, the enteric coating of the granules retained its integrity in the acid stage, as expected, and the percent release of omeprazole without the acid stage was not much different from the percent release of omeprazole with the acid stage. Figure 1 (b) and (c) show the percent of drug released from the omeprazole granules of one Cambodian sample and two Myanmar samples respectively. In these cases, the pattern of drug release in buffer without the acid stage was significantly higher than that with the acid stage, suggesting that the enteric coating of the failed samples was not fully effective.

### Acid degradation of omeprazole

**Fig. 2:**
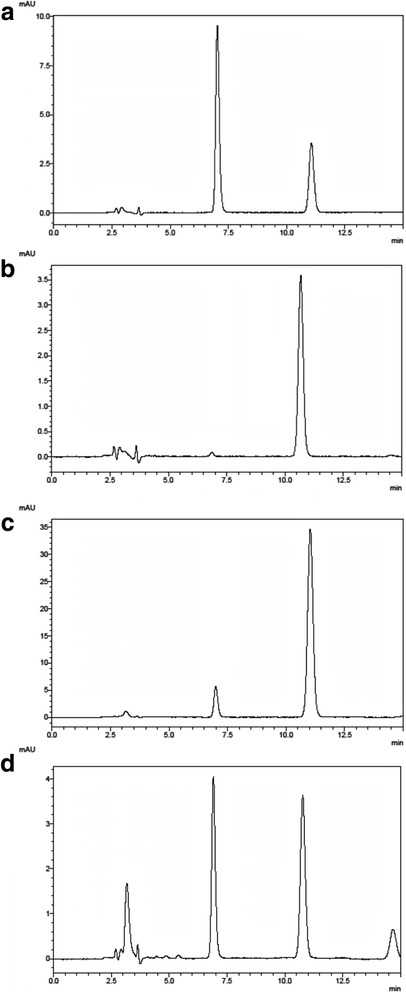
HPLC chromatogram of omeprazole samples in acid resistance stage. **a** Omeprazole reference standard; **b** standard omeprazole sample; **c** passed omeprazole sample; and **d** failed omeprazole sample

Typical chromatograms of reference standard omeprazole, standard omeprazole, a passed omeprazole sample and a failed omeprazole sample are shown in Fig. 2. The failed sample showed peaks indicating that degradation had occurred.

**Fig. 3:**
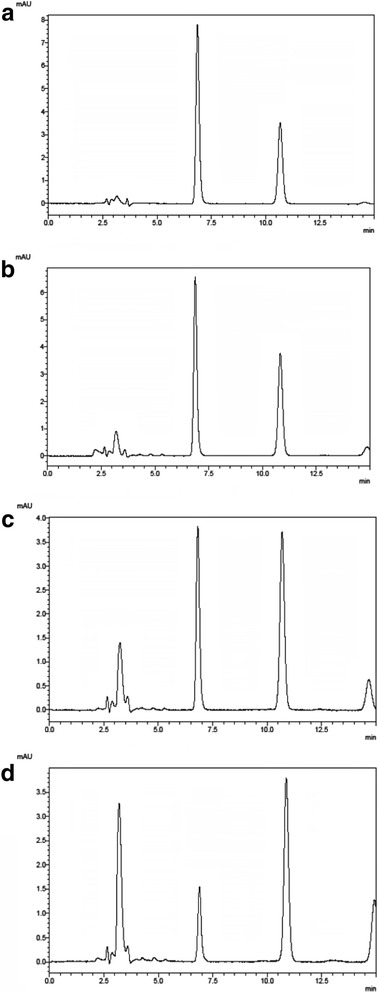
Chromatograms of pure omeprazole in acid at different time points. **a** 0 min; **b** 15 min; **c** 30 min; and **d** 45 min

**Fig. 4:**
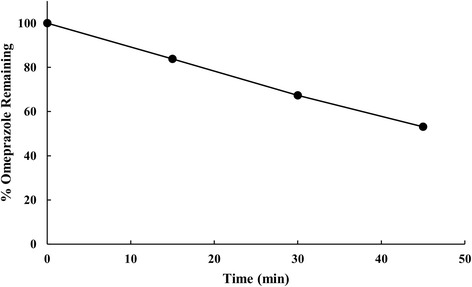
Time course of degradation of pure omeprazole in acid

To confirm this, reference standard (pure) omeprazole was exposed to acid (pH 4.5) and aliquots were withdrawn for HPLC analysis at 0, 10, 30, and 45 min. As shown in Fig. 3, peaks of degradation products increased time-dependently, and the time course of remaining omeprazole is shown in Fig. 4. These results are consistent with the conclusion that the failed omeprazole samples lacked effective enteric coating.

### Variability of granules in samples

**Fig. 5:**
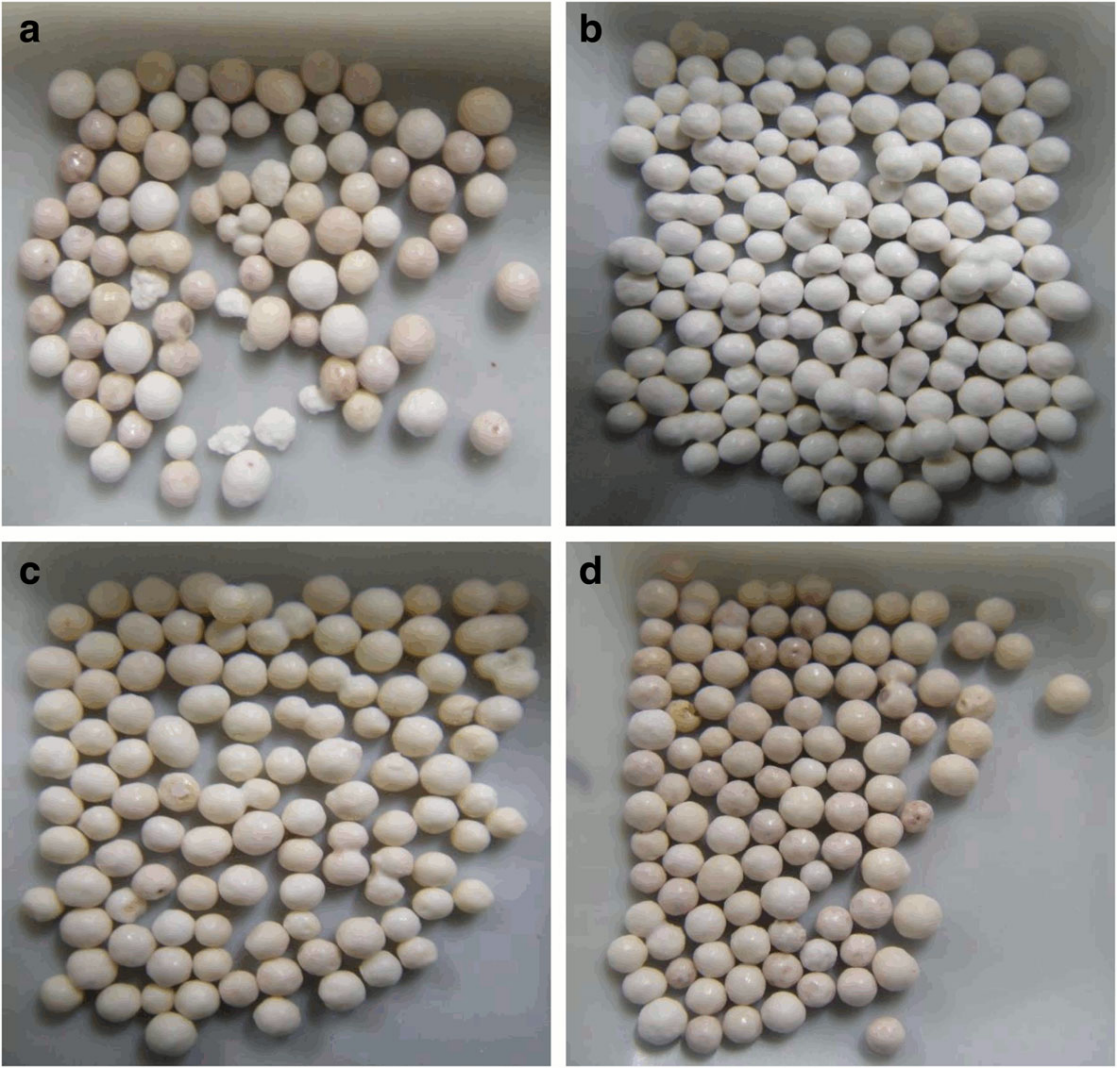
Difference in the color of granules in a capsule (sample A-078). **a** Mixed granules found after opening the capsule shells; **b** separated white granules; **c** grey granules; and **d** yellow granules

Considerable variability related to the formulation or manufacturing process of omeprazole has been suggested in Cambodian samples [6], and we found similar variation in some Myanmar samples. The shape of the granules ranged from spherical to irregular, and different-colored granules were seen, as illustrated in Fig. 5. We isolated white, yellow, and grey granules, and quantified them individually. The omeprazole contents in the white, grey and yellow granules were 64.1 ± 0.2, 61.4 ± 0.5 and 42.7 ± 1.7 mg (mean ± SD) per 267.8 mg of granules (average weight of six granules).

### Examination of granules by scanning electron microscopy

**Fig. 6:**
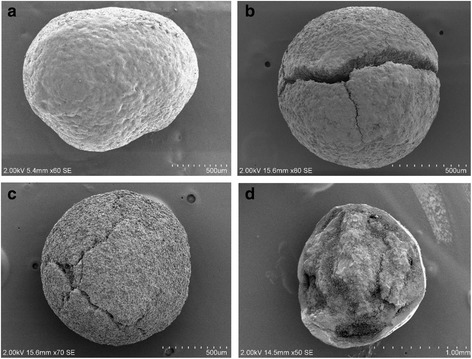
SEM images of cracked and fractured pellets found in two representative Cambodian samples. **a** Standard sample; **b** and **c** two different granules of sample A-063; and **d** Sample A-108

Representative SEM images are shown in Fig. 6. Compared with the standard sample (a), granules from failed omeprazole samples collected in Cambodia in 2010 showed cracks in the enteric coating, along with the broken pellets. These were mixed with the regular granules in the capsule.

### Examination of granules by X-ray computed tomography

**Fig. 7:**
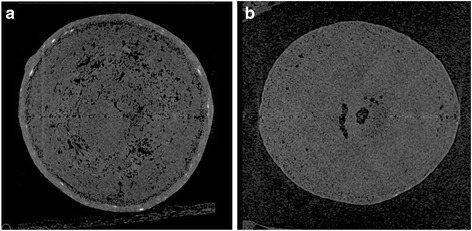
X-Ray CT images of granules found in sample B-040 collected in Cambodia in 2010. Note the presence of an apparently intact enteric-coated layer in (**a**) and the absence of an enteric-coated layer in (**b**)

**Fig. 8:**
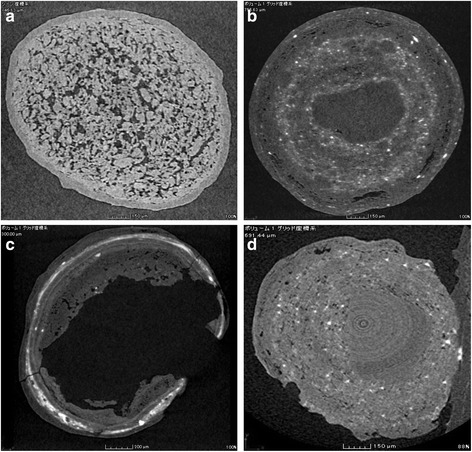
X-Ray CT images of granules found in sample A-078 collected in Myanmar in 2014. **a** Standard sample; **b** regular granule with apparently intact coating (white granule 1); **c** irregular granule with hole (white granule 2); and **d** yellow granule with incomplete coating

To confirm the absence of functional enteric coating, we performed X-ray CT on selected samples that failed severely in the dissolution test and conducted a comparative study of the standard sample and failed samples. Figure 7 shows two different granules taken from the same capsule in a sample collected in Cambodia in 2010. One granule shows an apparently intact coating (Fig. 7a), while the other (Fig. 7b) has essentially no coating at all. Similar results were seen in failed samples from Myanmar, which contained non-uniform granules, incomplete granules, and granules with holes. Figure 8 shows representative X-ray CT images of granules from a failed Myanmar sample.

## Discussion

Our findings for samples collected in Myanmar in 2014 indicate that the incidence and condition of substandard omeprazole medicines in Myanmar are quite similar to those in Cambodia. Yoshida et al. found that 45 out of 91 omeprazole samples collected in Cambodia in 2010 (49.5%) were unacceptable in the dissolution test (Table 1) [6]. Among the Myanmar samples, 23 (35.4%) failed the quantity test, although the extent of failure was generally marginal. A few failed due to over-content. Moreover, significant differences were observed in the quality test, where some brands passed in most cases, while others were consistently substandard. It was particularly noteworthy that the products of certain manufacturers failed consistently.

In the present work, our prime concern was the failures in dissolution tests. Among the 17 (26.2%) unacceptable samples from Myanmar, all failed in the buffer stage and 11 failed in both the acid and buffer stages. A key issue appeared to be that the coating of the granules did not provide good control of the drug release, so that rapid disintegration and dissolution occurred in the acid stage of the test, resulting in exposure of omeprazole to acid degradation [17, 19, 32, 33]. Indeed, an HPLC analysis showed that degradation of omeprazole in the granules of failed samples during the acid stage was similar to that of pure omeprazole (Fig. 3), confirming that the enteric coating was ineffective.

This conclusion was further supported by SEM images (Fig. 6), which revealed fractured pellets and pellets with incomplete coating, together with pellets with apparently intact coating. Macroscopically, a sample from Cambodia contained two different types of granules in a single capsule, and the X-ray CT images showed that one type of granule lacked enteric coating (Fig. 7). Similar results were found in a sample from Myanmar, which appeared to contain three different types of granules (Fig. 5). The X-ray CT images revealed that the coating of some granules was incomplete and some granules contained holes (Fig. 8). Thus, there was marked inconsistency among omeprazole granules in capsules, and this suggests that at least some manufacturers were using inadequate enteric coating technology or conditions. However, we were unable to confirm this with the manufacturers. Substandard medicines are a serious public health issue. In the case of omeprazole, substandard samples without enteric coating or with incomplete enteric coating will degrade rapidly in the acidic environment of the stomach after oral administration, and this may result in treatment failure.

It should be noted that in some cases the size of our samples was insufficient for detailed examination, and this represents a weakness of our study, in that we could not fully assess the actual extent of quality failure in our analysis. Another limitation is that; we could not confirm the coating material used during the manufacture of the enteric coated pellets or if there was inadequate coating method (e.g. inadequate equipment or inadequate coating parameters) as the response to the questionnaire from the manufacturers was minimum. Therefore, further investigation is needed to establish precisely the scale of the problem of substandard medicines in Myanmar and Cambodia. In addition, action, including regulatory measures, should be initiated to prevent the manufacture and sale of substandard medicines.

## Conclusions

Samples of omeprazole capsules collected in Cambodia in 2010 and Myanmar in 2014 showed high failure rates in pharmacopoeial testing, especially in the dissolution test. In-vitro dissolution profiling, scanning electron microscopy and X-ray computed tomography showed that failed samples contained granules with ineffective (cracked or incomplete) or absent enteric coating. This would result in premature dissolution in acidic conditions after oral administration, and could result in treatment failure. This situation is a potentially serious public health issue that needs to be addressed by regulatory authorities in Cambodia and Myanmar, possibly through legal measures and collaborative initiatives with manufacturers.

## Abbreviations

BP: British Pharmacopoeia
DDF: Department of Drugs and Food
FDA: Food and Drug Administration
HPLC: High-performance liquid chromatography
JPMA: Japan Pharmaceutical Manufacturers Association
NF: National Formulary
SEM: Scanning electron microscopy
USP: United States Pharmacopoeia
WHO: World Health Organization
X-Ray CT: X-Ray computed tomography
